# Gut Microbiota Profiling by 16S rRNA Sequencing in Children with Mild or Intermittent Asthma: A Pilot Study in Alessandria, Italy

**DOI:** 10.3390/microorganisms13122664

**Published:** 2025-11-24

**Authors:** Valentina Pizzo, Alessia Francese, Paolo Bottino, Carlotta Bertolina, Franca Gotta, Michela Gandino, Enrico Felici, Riccardo Mazzucco, Marinella Bertolotti, Annalisa Roveta, Andrea Rocchetti, Antonio Maconi

**Affiliations:** 1 Microbiology and Virology Laboratory, Azienda Ospedaliero-Universitaria SS. Antonio e Biagio e Cesare Arrigo, 15121 Alessandria, Italy; valentina.pizzo@ospedale.al.it (V.P.); paolo.bottino@ospedale.al.it (P.B.); fgotta@ospedale.al.it (F.G.); arocchetti@ospedale.al.it (A.R.); 2 Research Laboratories, Research and Innovation Department, Azienda Ospedaliero-Universitaria SS. Antonio e Biagio e Cesare Arrigo, 15121 Alessandria, Italy; aroveta@ospedale.al.it; 3 Research Training Innovation Infrastructure, Research and Innovation Department, Azienda Ospedaliero-Universitaria SS. Antonio e Biagio e Cesare Arrigo, 15121 Alessandria, Italy; mbertolotti@ospedale.al.it (M.B.); amaconi@ospedale.al.it (A.M.); 4 Pediatric and Pediatric Emergency Unit, Children Hospital, Azienda Ospedaliero-Universitaria SS. Antonio e Biagio e Cesare Arrigo, 15121 Alessandria, Italy; mgandino@ospedale.al.it (M.G.); enrico.felici@ospedale.al.it (E.F.); 5 Research and Innovation Department, Azienda Sanitaria Locale, 15121 Alessandria, Italy; rmazzucco@aslal.it

**Keywords:** pediatric asthma, gut microbiota, dysbiosis, iSeq sequencing, mediterranean diet

## Abstract

Asthma is one of the most common chronic diseases in childhood, and the gut microbiota is increasingly recognized as a key player in its pathogenesis through the gut–lung axis. This pilot, cross-sectional study analyzed the intestinal microbiota of 20 children (6–9 years) with intermittent or mild persistent asthma, in the absence of a healthy control group. Stool samples were processed with 16S rRNA sequencing (Illumina iSeq100, V3–V6 regions) and analyzed with MicrobAT-SmartSeq software. Contrary to most literature, no enrichment of *Proteobacteria* was detected, and *Faecalibacterium* was not reduced but emerged as the most abundant genus. Higher adherence to the Mediterranean diet correlated with greater microbial richness, while asthma phenotypes showed no major taxonomic differences. Pharmacological treatments modulated microbial profiles: leukotriene antagonists correlated with protective taxa, whereas inhaled corticosteroids and bronchodilators were linked to reduced beneficial functions. These findings highlight an overall eubiotic microbiota in asthmatic schoolchildren and call for larger, controlled studies to confirm these novel observations.

## 1. Introduction

The gut microbiota is a key modulator of immune homeostasis, especially during early life. Its composition is influenced by various factors including birth mode, feeding practices, antibiotic exposure, and dietary patterns. The gut–lung axis, a bidirectional communication system linking gut microbiota to respiratory health, has been implicated in the modulation of asthma through immune regulation and microbial metabolite signaling [1]. Specific microbial taxa, such as *Faecalibacterium prausnitzii*, *Bifidobacterium* spp., and *Akkermansia muciniphila*, have been associated with anti-inflammatory effects and reduced asthma risk. However, the microbiota composition in school-age children with asthma remains under-investigated, particularly in relation to lifestyle and dietary habits [2,3,4].

Asthma is one of the most prevalent non-communicable chronic diseases globally, affecting around 10% of the pediatric population worldwide [5].

It is defined by the Global Initiative for Asthma (GINA) as a heterogeneous disease characterized by chronic inflammation of the airways, with respiratory symptoms such as wheezing, shortness of breath, breathlessness, chest tightness, and coughing, which vary in frequency and intensity [6]. While the precise pathogenesis of asthma remains unclear, it is believed to be influenced by several factors during childhood, including neonatal antibiotic use, mode of delivery, maternal diet, and formula feeding.

Alterations in gut microbial composition, known as dysbiosis, have been associated with immune dysregulation and increased asthma susceptibility. While most studies have focused on the first year of life as a critical period for microbiota maturation and immune programming, the gut microbiota continues to evolve throughout childhood and may still influence respiratory outcomes [7,8]. The interaction between diet, pharmacological treatments, and microbiota thus represents a key area of investigation in pediatric asthma [9].

Alterations in gut microbial composition have been associated with an increased risk of asthma, allergies, and other immune-mediated disorders, suggesting that gut dysbiosis may be a key environmental factor in the transition from health to disease [10].

The gut microbiota of children undergoes continuous development throughout childhood and adolescence, differing in composition and abundance from that of adults. In particular, the pediatric gut microbiota is characterized by a predominance of *Bifidobacterium* spp., *Faecalibacterium* spp., and members of the *Lachnospiraceae* family, whereas the adult microbiota is more enriched in *Bacteroides* spp. [11].

Both the intestinal and respiratory microbiota play a central role in immune regulation. In asthma, environmental and lifestyle factors can influence these microbial ecosystems, and imbalances between commensal and pathogenic taxa may promote airway inflammation. Although the causal direction remains uncertain, growing evidence supports the existence of a gut–lung axis, through which intestinal dysbiosis can influence pulmonary immune responses and potentially contribute to asthma pathogenesis [12,13,14].

Despite the increasing number of studies worldwide, no research to date has characterized the gut microbiota of Italian children with mild or intermittent asthma. Most available data refer to early infancy or to moderate-to-severe asthma phenotypes in different populations. Addressing this gap is essential to better understand how dietary patterns and pharmacological treatments may shape the intestinal microbiota in children with less severe forms of asthma.

Accordingly, this pilot study aimed to evaluate the gut microbiota composition in school-aged children diagnosed with asthma, to identify potential microbial alterations or characteristic signatures associated with the disease. A secondary objective was to explore the relationship between gut microbiota profiles and lifestyle factors, including diet, physical activity, and medication use. Dietary habits were assessed using the KIDMED (Mediterranean Diet Quality Index in children and adolescents) questionnaire [15], which measures adherence to the Mediterranean diet, a dietary pattern known for its anti-inflammatory properties and beneficial impact on the microbiota.

Understanding the interactions between microbiota, lifestyle, and pediatric asthma may contribute to the identification of novel preventive or therapeutic targets based on microbiome modulation [16,17].

## 2. Materials and Methods

Between 13 June 2024, and 24 October 2024, a total of 21 pediatric patients were enrolled in the study, with one participant subsequently withdrawing consent. The study population included children aged 6 to 9 years with a confirmed diagnosis of bronchial asthma, all receiving care at the Pediatric Hospital of Alessandria (AOU AL). Asthma diagnosis and treatment classification followed the Global Initiative for Asthma (GINA) guidelines. Although GINA no longer distinguishes between “intermittent” and “persistent” asthma, patients were categorized according to treatment steps reflecting those clinical patterns. Children managed at Step 1 (short-acting β_2_-agonists as needed, without controller therapy) were considered to have intermittent asthma, while those at Step 2 (regular controller therapy with as-needed short-acting β_2_-agonists) were classified as having mild persistent asthma. The microbiological evaluation was carried out at the Microbiology Laboratory, Alessandria University Hospital. Ethical approval was obtained from the local ethics committee (approval code: “Aso.Ped.23.11”) on 15 April 2024, and written informed consent was collected from the legal guardians of each participant. For all enrolled patients, medical history was recorded, fecal samples were collected, and the KIDMED questionnaire was administered to assess dietary habits.

### 2.1. Sample Collection and Clinical Data

Fecal samples were collected at home by the children’s parents using the OMNIgene^®^ GUT OM-200 kit (DNAgenotek^TM^, Stittsville, ON, Canada), which is specifically validated to preserve nucleic acids and microbial composition at room temperature for extended periods (up to 60 days). This device contains a stabilization medium that maintains DNA integrity without refrigeration, allowing convenient home collection and reliable microbial preservation. Samples were then stored at room temperature until NGS analysis. The collection process was assisted by pediatricians and followed a defined transport route to the Microbiology and Virology Lab of AOU Alessandria.

All clinical data, including demographics, asthma classification, treatment, infection history, type of delivery, allergies and antibiotic use, were retrieved from medical records.

Information on pharmacological treatments was obtained through the clinical questionnaire, which recorded the type of medication used for asthma management, including bronchodilators, leukotriene receptor antagonists, inhaled corticosteroids, antihistamines, or other drugs. Data on treatment duration or time since last administration were not collected in detail, as the study design focused primarily on the presence or absence of these therapeutic categories. Regarding antibiotic exposure, data were systematically collected through a parental questionnaire. Current antibiotic therapy represented an exclusion criterion for participation, and no child was receiving antibiotics at the time of stool collection. Previous antibiotic use was reported as occurring 2–3 months, 6 months, or more than 9 months before sampling. No participant reported probiotic supplementation in the four weeks preceding sample collection.

Moreover, a KIDMED questionnaire was administered to assess adherence to the Mediterranean diet and additional information on physical activity was also requested. The KIDMED questionnaire was completed by the child’s parent or legal guardian, under the supervision of a pediatrician, to ensure accuracy and consistency of responses. All data were loaded into a dedicated electronic data capture system, REDCap (Research Electronic Data Capture) [18,19].

### 2.2. DNA Extraction and 16S rRNA Sequencing

Bacterial DNA was extracted using the QiAmp^®^ PowerFecal^®^ Pro DNA Kit (Qiagen, Hilden, Germany) with an automated extractor. As suggested by the device manufacturer, a pre-treatment was carried out with an initial volume of 250 µL, transferred into the PowerBead Pro Tube of the extraction kit. 800 µL of Solution CD1 were added and, after gentle vortexing, the QIAamp PowerFecal Pro DNA Kit protocol was started from step 2. Elution was performed in 100 µL and DNA quantification was performed using a Qubit fluorometer (Thermofisher, Waltham, WA, USA), in order to dilute samples until a standardized concentration of 1 ng/µL.

NGS analysis was based on the evaluation of the V3–V6 hypervariable regions of the 16S rRNA gene, amplified using the Amplimix V3–V6 solution A (Arrow Diagnostics, Genova, Italy), according to the manufacturer’s instructions. Sequencing libraries were prepared with specific indices and pooled at a final concentration of 20 nM. After dilution to 120 pM, libraries were sequenced onto the iSeq-100 platform (Illumina^®^, San Diego, CA, USA).

### 2.3. Bioinformatics and Statistical Analysis

Raw data were denoised, trimmed and classified using the MicrobAT software version 1.3.0 (SmartSeq srl, Novara, Italy). After quality control and filtering, sequencing produced an average of approximately 110,000 reads per sample (range 85,000–150,000), with an average read length of ~420 bp. Rarefaction analysis confirmed that all samples reached a plateau, indicating > 97% coverage and sufficient sequencing depth for downstream analyses. Unclassified sequences were excluded from α- and β-diversity and correlation analyses. Due to an average 45% unclassified sequences, taxonomic analysis was conducted until the genus level. Pathogenic genera (e.g., Proteobacteria) were highlighted in red, while beneficial or eubiotic-associated genera (e.g., Firmicutes, Bacteroidetes, Actinobacteria) were indicated in blue or green.

Functional predictions were generated automatically by the MicrobAT platform, which classifies bacterial taxa into 13 predefined metabolic categories (e.g., carbohydrate metabolism, amino acid metabolism, SCFA production, oxidative stress response). These categories are based on curated reference databases linking specific bacterial taxa to known metabolic pathways described in the literature. The resulting Z-scores represent predicted metabolic potentials derived from 16S rRNA-based taxonomic profiles rather than direct metagenomic quantification.

Categorical variables were expressed as frequencies and percentages; continuous data were reported as median and interquartile range (IQR).

These metrics were used to evaluate microbial community stability and potential dysbiosis in asthmatic children. Results were compared towards abovementioned collected variables and using the Kruskal–Wallis and Mann–Whitney U tests.

Heatmaps were generated to visualize the distribution and clustering of some clinical and lifestyle variables across subjects. Variables included in the analysis were selected based on their potential influence on gut microbiota composition, such as dietary adherence (KIDMED score), pharmacological treatments, asthma phenotype, and physical activity. Data were previously normalized using z-score transformation. Hierarchical clustering was performed using the Manhattan distance metric and complete linkage method. The resulting dendrograms illustrate the inter-individual similarity patterns based on the multivariate profiles.

In order to assess if there were any differences in the within-group (intra-subject) variability, the homogeneity of multivariate dispersions (i.e., the distance of individual samples to their group centroid) was tested by means of the betadisper function from the vegan package. This procedure was used to test whether the observed among-group variation arises from differences in within-group dispersion rather than from shifts in group centroids in the multivariate space. The significance of dispersion differences between groups was further assessed by permutation test.

All tests were two-tailed, and a *p*-value < 0.05 was considered statistically significant. For multiple comparisons, Bonferroni correction was applied, and adjusted *p*-values are reported where appropriate. Effect sizes (Pearson’s *r*) and corresponding confidence intervals were calculated for correlation analyses.

All statistical analysis was performed throughout the use of R Studio (version 4.2.1).

#### Microbiota Composition Evaluation

α-Diversity and β-diversity tests were used to assess the microbiota composition on enrolled children.

With regard to α-diversity, the following indexes were used: Shannon Index, Chao1 Index, Simpson Index (D), and Pielou Index. Instead, for β diversity, the following metrics were performed: Bray–Curtis, Manhattan and Jaccard. Statistical evaluation of β-diversity was assessed throughout the Permanova analysis. Again, obtained results were evaluated towards collected variables and all tests were two-tailed. A *p*-value < 0.05 was considered statistically significant. For multiple comparisons, Bonferroni correction was applied, and adjusted *p*-values are reported where appropriate. Effect sizes (Pearson’s *r*) and corresponding confidence intervals were calculated for correlation analyses.

### 2.4. Raw Percent Data

Among the functions of the MicrobAT software is the determination of relative abundance percentages for the 13 most represented bacterial categories, reported together with their reference ranges. These ranges, however, are derived from cohorts of healthy adults and were therefore not applicable to the present study.

The 13 bacterial categories examined were:

- Beneficial bacteria;
- Butyrate-producing bacteria;
- Lactic acid-producing bacteria;
- Probiotic bacteria;
- Oral bacteria;
- Intestinal mucosa–specific bacteria;
- LPS-producing bacteria;
- Sulfate-reducing bacteria;
- Glutamate-producing bacteria;
- Anti-inflammatory bacteria;
- Acetate-producing bacteria;
- Propionate-producing bacteria;
- Proteolytic bacteria.

Using an Excel spreadsheet, the following parameters were calculated for each metabolic function: mean, standard deviation, and Z-score.

On normalized data (Z-scores), cluster analysis was performed using Excel’s Analysis ToolPak (K-means clustering), identifying k = 3 as the optimal number of clusters.

Distribution of subjects across clusters:

- Cluster 1: 8 subjects;
- Cluster 2: 6 subjects;
- Cluster 3: 6 subjects.

The division into three groups suggests the existence of different phenotypes of gut microbiota alteration in asthmatic children. The clustering technique employed represents each cluster by its centroid, i.e., the mean vector of the variables within the cluster. The centroids are reported in Table 1.

The clusters identified on the basis of similarity in metabolic functions were as follows:

1. Protective profile, with almost all Z-scores > 0 (e.g., beneficial bacteria +0.805; butyrate producers +0.844), characterized by high abundance of beneficial bacteria and anti-inflammatory metabolites, indicating a favorable microbiota.
2. Pro-inflammatory profile, with Z-scores < 0 (e.g., beneficial bacteria −0.486; butyrate producers −1.017), in which most protective functions were reduced, suggesting substantial impairment of microbial functionality.
3. Intermediate profile, a mix of positive and negative Z-scores, with a combination of preserved and compromised components. Although many functions showed negative values, lactic acid producers (+0.342) and glutamate producers (+0.617) were positive, representing a more heterogeneous profile.

## 3. Results

Twenty pediatric patients with a median age of 8.13 (6.65–8.48) were enrolled, 75% of whom were male. Most patients were diagnosed with intermittent asthma (65%), the remaining with mild persistent asthma. The main therapies included: bronchodilators (70% of all patients), antileukotrienes (50%), inhaled corticosteroids (75%), and antihistamines (15%).

Regarding antibiotic administration, 60% of patients had not taken antibiotics for more than 9 months, 20% for 6 months, and a further 20% for about 3 months.

Simple spirometry was performed on 17 patients; it showed a median of 1.62 (1.36–1.80) L for Forced Expiratory Volume in 1 s (FEV 1), 1.76 (1.52–1.92) L for (Forced Vital Capacity) FVC and 92.00 (87.00–96.00) L for the FEV1/FVC ratio.

Eosinophil count analysis was evaluated in 13 patients, with a median value of 190.00 (130.00–610.00).

80% of subjects exercised at least once a week. About diet, the results of the KIDMED questionnaire showed that 25% adhered closely to the Mediterranean diet and 60% adhered moderately.

To assess α diversity, bacterial abundance was compared by considering physical activity, corticosteroid therapy, and Mediterranean diet adherence. As regards bacterial abundance among children who exercised compared to those who did not, no statistically significant differences were found between the two groups in terms of bacterial abundance (Shannon index *p* = 0.40; Simpson index *p* = 0.70), as well as between those who took or did not take corticosteroids (Shannon index *p* = 0.46; Simpson index *p* = 0.69) or between those who adhered or did not adhere to the Mediterranean diet (Shannon index *p* = 0.17; Simpson index *p* = 0.40).

### 3.1. Alpha Diversity of Genera and Most Representative Variables

**Figure 1:**
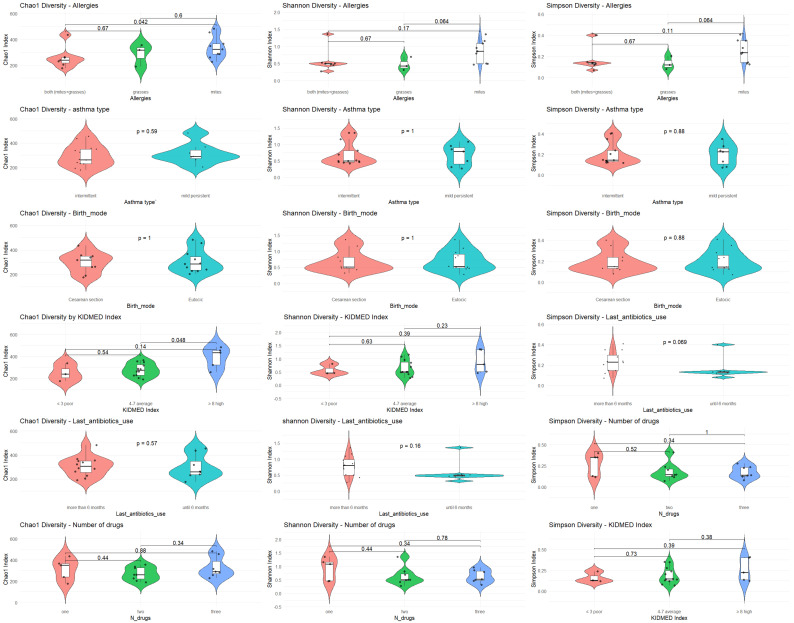
α diversity in relation to key clinical and environmental variables.

α diversity was analyzed with respect to the most significant variables: allergies, asthma type, mode of delivery, KIDMED score, antibiotic use, and type of medication. The analysis was performed using Chao1, Shannon, and Simpson diversity indices (Figure 1).

### 3.2. Beta Diversity of Genera and Most Representative Variables

**Figure 2:**
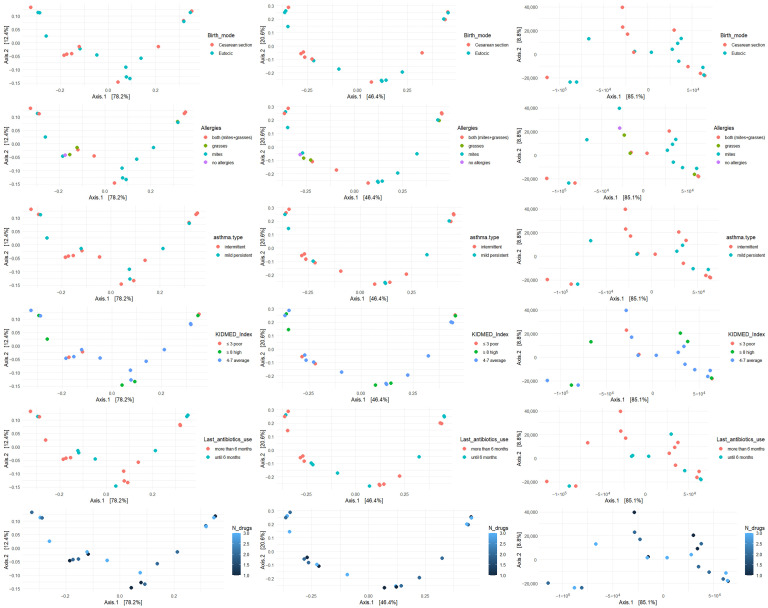
β diversity of bacterial genera across key clinical and environmental variable.

**Figure 3:**
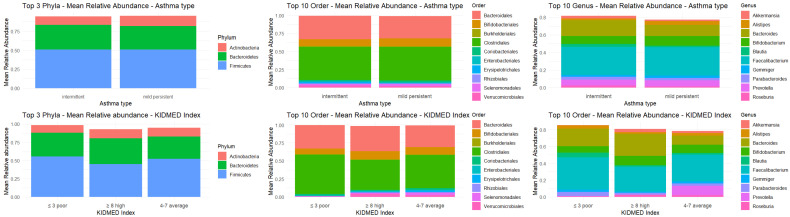
Mean relative abundances of phyla, order, and genus.

**Figure 4:**
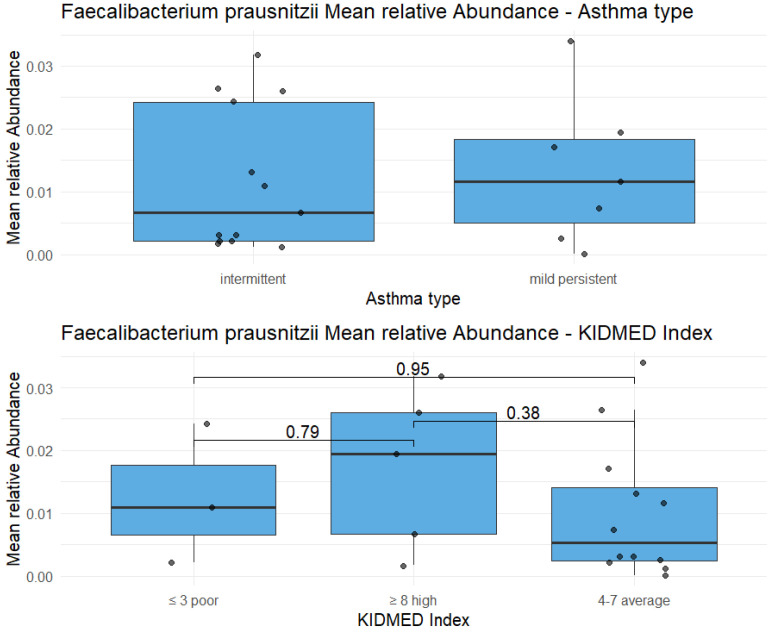
Highlight on *Faecalibacterium prausnitzii*: the relative abundance of this species doesn’t show statistically significant differences between two types of asthma (*p*-value = 0.72), as well as between three KIDMED scores (*p*-value in the figure).

β diversity was analyzed with respect to the most significant variables: allergies, asthma type, mode of delivery, KIDMED score, antibiotic use, and number of medications taken. The analysis was performed using Bray–Curtis, Jaccard, and Manhattan metrics (Figure 2, Figure 3 and Figure 4).

### 3.3. Relative Abundances

*Firmicutes* represented the most abundant phylum. *Bifidobacteriales*, an order characteristic of early childhood, were still well represented despite the age of study participants reaching up to 9 years, particularly in the group with higher KIDMED Index scores. At the genus level, *Faecalibacterium* showed a markedly higher relative abundance compared to all other genera.

### 3.4. Genera: Metabolic Categories and Cluster Analysis

Analysis of raw data showed that, within the microbiota of the study subjects, the most represented beneficial bacterial genera were *Faecalibacterium* (17.7%), *Bacteroides* (9.3%), *Bifidobacterium* (5.5%), *Blautia* (1.4%), and *Roseburia* (1.1%), whereas the pathogenic component—Clostridium IV (0.29%), *Sutterella* (0.26%), and Escherichia-Shigella (0.25%)—was virtually absent.

### 3.5. Metagenomic Analysis: Comparative Taxonomic Profiles

As part of the metagenomic analysis of fecal samples, Venn diagrams were used to visualize and compare microbial taxa present in subgroups of asthmatic subjects, stratified according to two variables of interest: clinical phenotype of asthma and adherence level to the Mediterranean diet. The diagrams were constructed based on the identification of microbial genera shared across groups and those exclusive to each, in order to highlight both the common and condition-specific microbial components.

**Figure 5:**
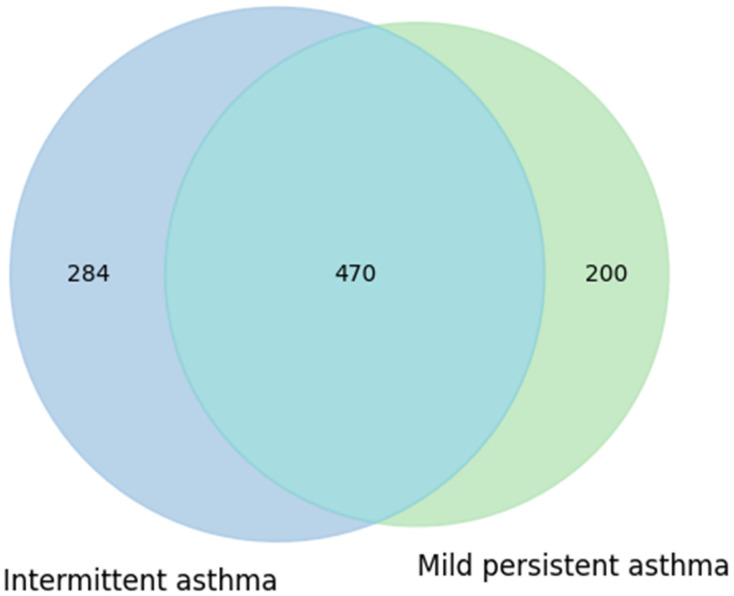
Similarities and own particular characteristics of taxa (taxonomic level: genus) between intermittent and mild persistent asthma groups: both types of asthma share 470 taxa. Intermittent asthma has 274 unique taxa, and persistent asthma has 200 unique ones, thus showing a balanced situation.

A first comparison was carried out between children with intermittent asthma and those with mild-to-moderate persistent asthma. Data were extracted from RedCap: of the 20 children, 12 had mild persistent asthma and 8 had intermittent asthma. Taxonomic data were obtained from MicrobAT to identify the microbial components common to and specific for each condition. The results of this analysis are presented in Figure 5 as a Venn diagram.

The diagram shows that the intermittent asthma group comprised 754 taxa, while the mild persistent asthma group comprised 670 taxa. The two groups shared a substantial number of taxa (470), with 284 taxa unique to the intermittent group and 200 taxa unique to the mild persistent group (Figure 5). This partial overlap suggests that, at the presence/absence level, no marked differences in taxonomic composition were observed between the two phenotypes. These results may indicate that the clinical characteristics of asthma do not substantially influence the variety of taxa present, although potential differences in relative abundance or metabolic functionality cannot be excluded.

A second comparison was carried out considering dietary patterns, stratifying subjects into three groups according to their level of adherence to the Mediterranean diet: low (3 children), medium (12 children), and high (5 children).

The high KIDMED score group comprised 619 taxa, the medium group 752, and the low group only 359, substantially fewer than the other two. The number of taxa shared across all three groups were 278, while the degree of overlap between specific pairs of groups varied considerably:

- Medium + High adherence: 169 shared taxa;
- Medium + Low adherence: 31 shared taxa;
- High + Low adherence: 20 shared taxa.

The number of taxa unique to each group also differed significantly:

- Medium adherence: 274 unique taxa;
- High adherence: 152 unique taxa;
- Low adherence: 30 unique taxa.

**Figure 6:**
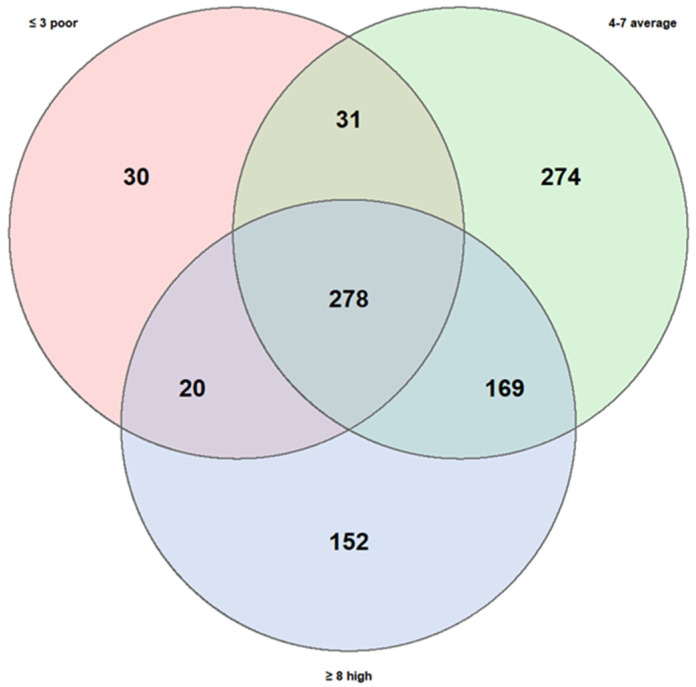
Similarities and own particular characteristics of taxa (taxonomic level: genus) between low, medium, and high adherence to the Mediterranean diet. The three KIDMED scores share 278 taxa. The high score has 152 unique taxa; the medium score has 274 unique and the low score has 30 unique; low score shows notable poverty of biodiversity.

These results (Figure 6) suggest that adherence to the Mediterranean diet may exert a stronger influence on gut microbiota diversity compared to the variability observed between asthma phenotypes. In particular, low adherence appeared to be associated with reduced richness of unique taxa, whereas the medium- and high-adherence groups showed more diverse taxonomic profiles, potentially indicative of greater microbial stability and resilience.

**Figure 7:**
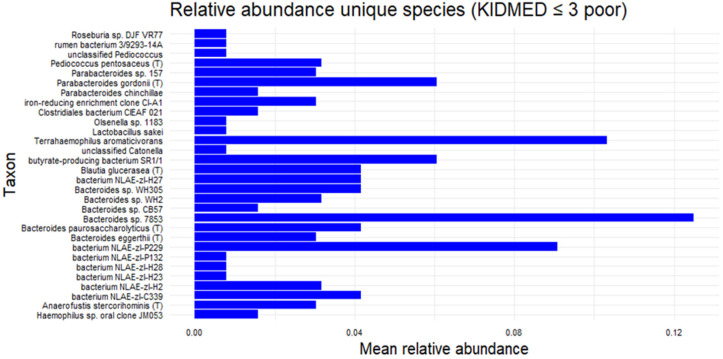
Exclusive bacterial species in the subgroup with KIDMED ≤ 3.

A subsequent analysis focusing on the subgroup with KIDMED scores ≤ 3 identified several exclusive bacterial species (shown in the Venn diagram of Figure 6), although their relative abundances were generally low. Among these, *Bacteroides* sp. 78–53 exhibited the highest mean relative abundance (>0.12), followed by *Terrahaemophilus aromaticivorans* (~0.11), *Bacterium* NLAE-ZLP229 (~0.09), and *Parabacteroides gordonii* T (~0.06). The presence of *Haemophilus* sp. oral clone was also noted. (Figure 7) at present, these findings remain descriptive.

### 3.6. HEATMAP

**Figure 8:**
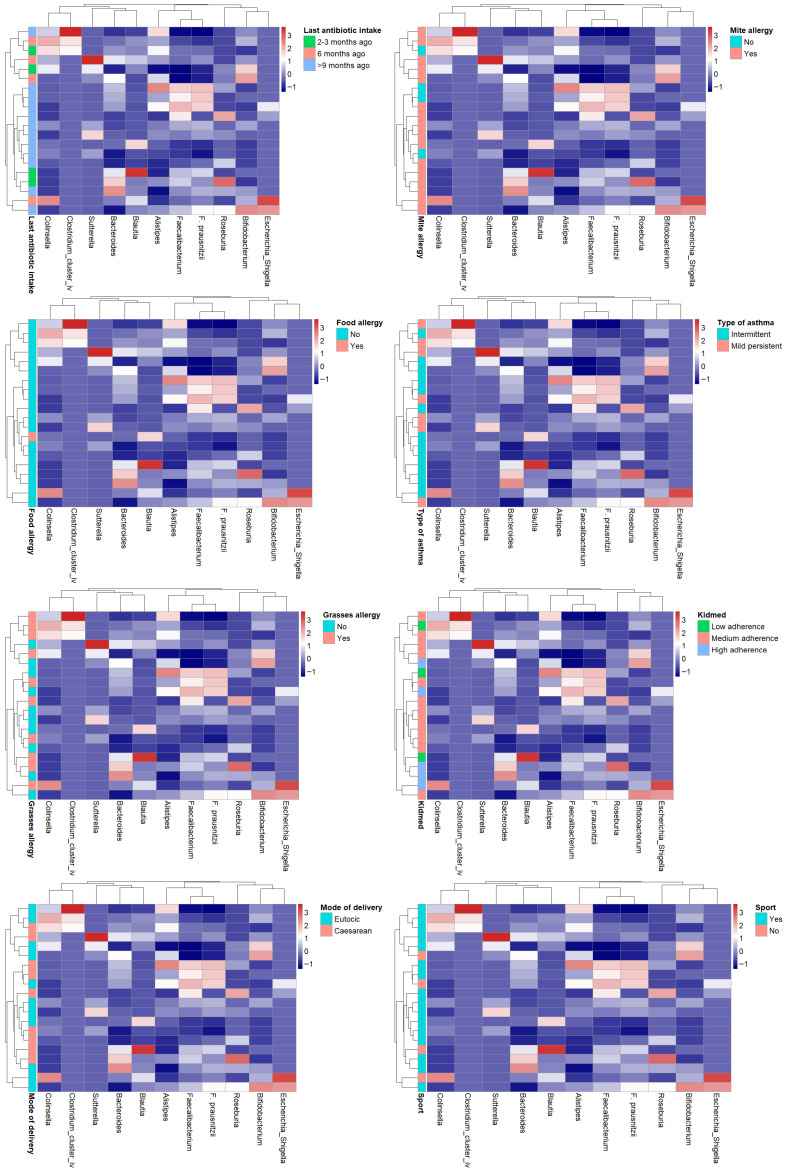
Heatmap showing the relative abundances (z-score normalized) of dominant gut bacterial genera across subjects. Rows correspond to individual participants and columns to bacterial taxa. Color intensity reflects relative abundance levels (blue = below mean, white = mean, red = above mean). Side annotations indicate participants’ sport activity group.

In order to identify potential common profiles acting as bacterial signatures, heatmaps were generated correlating bacterial genera of greatest interest for their anti-inflammatory roles (*Faecalibacterium*, *Bifidobacterium*, *Bacteroides*, *Roseburia*, *Blautia*) or pro-inflammatory roles (*Collinsella*, *Sutterella*, *Alistipes*, *Clostridium cluster IV*, *Escherichia*-*Shigella*) with the main variables collected in the study (KIDMED score, last antibiotic use, asthma type, physical activity, mode of delivery, and allergies to food, dust mites, and grasses). In the cohort of examined (intermittent vs. persistent asthma, adherence to the high vs. medium vs. low Mediterranean diet, yes vs. no sporting activity, etc.) no evident patterns of the microbiota emerge. (Figure 8).

### 3.7. Analysis of Pharmacological Use

To further explore potential relationships between gut microbiota composition and pharmacological treatment in asthmatic children, the following analyses were performed:

- Mean drug utilization for each cluster.
- Pearson correlation coefficients to measure the strength and direction of associations between metabolic functions and pharmacological treatments.
- The main (r, t) pairs derived from the most relevant correlations between metabolic and pharmacological variables.

Data on medication use were extracted from the RedCap platform. The five drug categories analyzed in this study were:

- Bronchodilators;
- Antileukotrienes;
- Inhaled corticosteroids;
- Antihistamines;
- Other.

For each subject, the Pearson correlation coefficient was calculated between:

- intervallo_x: the Z-score vector of the 13 metabolic functions.
- The binary vector (0/1) indicating the use of each of the five drug categories. The results of these correlations are summarized in Table 2.

As shown in Table 2, the conditional formatting obtained with Excel, with the following key:

- Red = strongly negative r;
- Yellow = r close to zero;
- Green = strongly positive r.

Inhaled corticosteroids exhibited the strongest negative correlations with protective functions (e.g., probiotics: r = −0.4435; mucosa-associated bacteria: r = −0.4452). Antileukotrienes correlated positively with bacteria involved in immune modulation, including LPS producers (r = 0.5047), known for their role in immune system education, as well as with intestinal mucosa–specific bacteria (r = 0.4626).

**Figure 9:**
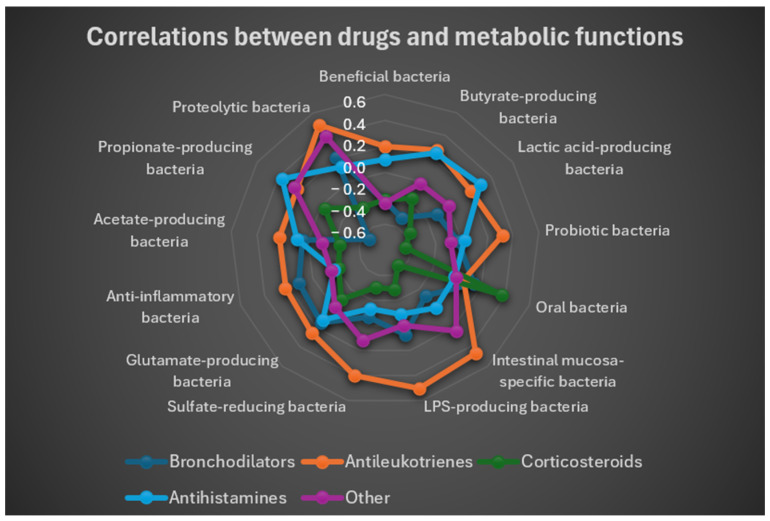
Radar plot based on the correlation coefficients between the use of the different drugs use and the metabolic functional groups. The values of the coefficient range from r = −0.6 to r = 0.6. Corticosteroids correlation with gut bacteria is negative, while that of the antileukotriens is positive.

To provide a concise and immediate visualization of correlations between drug use and functional groups of gut bacteria, a radar plot (Figure 9) was generated based on correlation coefficients. This plot allowed simultaneous observation of both positive and negative associations between each drug and bacterial functions, highlighting specific patterns. The radar plot showed that antileukotrienes tended to correlate positively with multiple groups of beneficial and metabolite-producing bacteria, whereas corticosteroids and bronchodilators displayed predominantly negative associations with these groups.

Overall, the results indicate a positive correlation between antileukotrienes and protective microbiota functions (beneficial bacteria, SCFA producers), while the use of inhaled corticosteroids and bronchodilators was associated with significant reductions in these functions.

Finally, differences in the within-group variability was also explored. The results indicated that multivariate dispersions were homogeneous across all tested factors (e.g., doing sport, last antibiotic assumption, type of asthma, type of delivery, mite allergy, grasses allergy), with non-significant *p*-values (all *p* > 0.05). These findings suggest that group variances were comparable and that between-group differences observed in subsequent analyses are unlikely to be driven by unequal within-group variability. Only the variable related to the presence of food allergies showed a statistically significant dispersion (*p* = 0.001), which was not further interpreted due to the presence of a single subject in one of the groups, making the estimate of within-group variance unreliable.

## 4. Discussion

This pilot study revealed several noteworthy findings regarding the role of diet and pharmacological treatments on the gut microbiota of children with asthma. Unexpectedly, the phylum Proteobacteria, which typically includes potentially pathogenic taxa, was almost completely absent.

This observation contrasts with previous reports in pediatric asthma cohorts where Proteobacteria, including genera such as *Streptococcus* and *Veillonella*, were relatively enriched [20].

Moreover, *Faecalibacterium*, contrary to our initial hypothesis, was not depleted but represented the most abundant genus overall. This aligns with published studies reporting *Faecalibacterium* as a hallmark of intestinal eubiosis and as a protective taxon frequently reduced in dysbiosis and allergic disease [21,22]. Its apparently lower abundance in the high KIDMED group was attributable to greater overall genus richness, suggesting a state of eubiosis.

Analysis of Mediterranean diet adherence demonstrated a significant association with taxonomic richness (Chao1 index), though not with Shannon or Simpson diversity indices. This indicates that dietary adherence primarily promotes microbial enrichment without substantially altering the balance among dominant taxa. Children with higher KIDMED scores harbored a broader range of taxa, including less represented species, reinforcing the potential protective role of this dietary pattern, in line with previous studies showing that Mediterranean diet adherence enriches SCFA-producing taxa and overall diversity [23,24].

Comparisons of taxonomic profiles between intermittent and mild persistent asthma phenotypes revealed substantial overlap, with only modest numbers of exclusive taxa, suggesting that asthma phenotype does not exert a major influence on the presence or absence of taxa [9]. By contrast, dietary adherence was associated with more pronounced differences: low adherence was linked to markedly reduced richness and few unique taxa, whereas medium- and high-adherence groups displayed richer and more diverse profiles, consistent with greater microbial stability and resilience.

Analysis of dendrogram heatmaps revealed an absence of evident patterns.

While this may partly be related to the limited sample size, which, in some cases, resulted in groups with very few individuals, the overall high within-group variability may also have contributed to the lack of distinct patterns. Although no significant differences in dispersion between groups were detected, the general degree of inter-individual variability appeared considerable. In addition, the influence of unmeasured or uncontrolled confounding factors cannot be excluded, which may have further obscured potential group-specific structures.

Pharmacological exposure also appeared to influence microbiota composition. Antileukotrienes correlated positively with protective functions, particularly short-chain fatty acid producers, whereas inhaled corticosteroids and bronchodilators were consistently associated with their reduction. At the genus level, children treated with corticosteroids exhibited lower median values for beneficial taxa, while untreated children showed preserved or even above-average levels. Unfavorable genera were generally scarce, although variability was greater among untreated subjects. Our data support the hypothesis that corticosteroids exert systemic effects on the gut microbiota. In agreement with this, Mahdavinia et al. reported that the gut microbiome of children with asthma and food allergy showed distinct signatures shaped by both clinical phenotype and treatment exposure [25].

Although our data indicate that dietary habits and pharmacological treatments appear to modulate the gut microbiota more markedly than asthma phenotype, this interpretation should be approached with caution. Given the descriptive nature of this pilot study and the limited sample size, the observed associations should be considered preliminary and hypothesis-generating rather than conclusive. Larger, controlled studies are required to confirm the relative contribution of each factor.

Moreover, the use of the term “eubiotic” in the present study is intended in a relative and descriptive sense, referring to the observed microbial equilibrium and the absence of dysbiotic features commonly reported in pediatric asthma cohorts (e.g., Proteobacteria enrichment or depletion of *Faecalibacterium*). While a general conceptual definition of eubiosis exists in the literature, standardized reference ranges and quantitative thresholds for identifying eubiosis in pediatric populations are still lacking. Therefore, the findings should be interpreted as exploratory observations rather than as evidence of a defined eubiotic state.

Taken together, these findings suggest that both dietary habits and pharmacological treatments play a critical role in shaping the gut microbiota of asthmatic children. Nevertheless, the study has several limitations: it was designed as a pilot investigation and included only 20 pediatric patients with asthma, with no healthy control group.

Another limitation is the lack of detailed information regarding treatment duration and time since the last administration of asthma medications. Pharmacological data were limited to the type of therapy used, which may have restricted the interpretation of potential associations between drug exposure and microbial composition. However, all treatments were consistent with the management of intermittent or mild persistent asthma, and none of the children were receiving systemic corticosteroids or antibiotics at the time of sampling.

Sequencing was performed using the Illumina^TM^ Iseq 100 platform with 16S methodology, which limited analytical depth and restricted resolution to the genus level. It should be noted that functional profiles were inferred from 16S rRNA data using the MicrobAT platform. Consequently, these results should be interpreted as predicted functional trends, and not as direct measurements of gene content or metabolic activity.

The small sample size, combined with the wide range of lifestyle variables analyzed and the lack of validated pediatric reference values, constrains the generalizability of our results. Future research, including the planned second phase with age-matched healthy controls and higher-resolution sequencing approaches, will be essential to validate these hypotheses and to clarify the role of diet and pharmacological treatments in reshaping the gut microbiota.

## 5. Conclusions

In this pilot study, children with mild or intermittent asthma displayed an overall eubiotic gut microbiota, characterized by the absence of Proteobacteria enrichment and high abundance of *Faecalibacterium*. Asthma phenotype did not significantly shape microbial diversity, whereas adherence to the Mediterranean diet and pharmacological treatments emerged as major modulators of taxonomic richness and functional profiles. These findings suggest that lifestyle and therapeutic factors may exert stronger effects on the pediatric gut microbiota than clinical phenotype alone. Larger, controlled studies with age-matched healthy cohorts and higher-resolution sequencing are needed to validate these results and clarify the potential for diet- and drug-related microbiome modulation in asthma management.

## Figures and Tables

**Table 1:** Centroids (mean Z-score values) of metabolic functions for each cluster.

Metabolic Function	Cluster 1	Cluster 2	Cluster 3	Overall
Beneficial bacteria	0.805496648	−0.485827572	−0.588167959	−5 × 10^−11^
Butyrate-producing bacteria	0.844105936	−1.016661229	−0.108813352	1 × 10^−10^
Lactic acid-producing bacteria	0.513701198	−1.027402397	0.342467466	0
Probiotic bacteria	0.873647455	−0.894559624	−0.270303649	1 × 10^−11^
Oral Bacteria	0.190498575	−0.04736572	−0.206632379	3 × 10^−10^
Intestinal mucosa-specific bacteria	0.69149407	−0.811753908	−0.110238185	−1.5 × 10^−10^
LPS-producing bacteria	0.923484745	−0.822989653	−0.408323341	−2.5 × 10^−10^
Sulfate-reducing bacteria	0.824768242	−0.927681718	−0.172009271	0
Glutamate-producing bacteria	0.200418197	−0.884421212	0.617196948	−5 × 10^−11^
Anti-inflammatory bacteria	0.89242426	−0.65565864	−0.534240374	−1 × 10^−10^
Acetate-producing bacteria	0.771341253	−1.034943448	0.006488445	5 × 10^−11^
Propionate-producing bacteria	0.603046162	−0.685208472	−0.118853078	0
Proteolytic bacteria	0.641295235	−1.07647622	0.221415907	−1.5 × 10^−10^

**Table 2:** Correlations between metabolic functions and pharmacological treatments.

Metabolic Function	Bronchodilators	Antileukotrienes	Corticosteroids	Antihistamines	Other
Beneficial bacteria	−0.223190801	0.204655993	−0.20905456	0.09928262	−0.234841045
Butyrate-producing bacteria	−0.316955926	0.271690621	−0.14801942	0.246965127	−0.019756754
Lactic acid-producing bacteria	−0.115010926	0.210818511	−0.36514837	0.295204962	3.27 × 10^−20^
Probiotic bacteria	−0.02800096	0.320042373	−0.44354059	0.01808443	−0.08812258
Oral bacteria	0.138477629	0.048879561	0.366704563	−0.020811271	−0.011148733
Intestinal mucosa-specific bacteria	−0.127891769	0.462689774	−0.44522344	−0.004319302	0.233526291
LPS-producing bacteria	0.069788146	0.504682704	−0.29145537	−0.088950853	−0.000335159
Sulfate-reducing bacteria	−0.069311897	0.401755699	−0.30528753	−0.133268344	0.121233276
Glutamate-producing bacteria	0.145757887	0.256830622	−0.08899957	0.122230961	−0.011869967
Anti-inflammatory bacteria	0.114172596	0.224230528	−0.21576593	−0.177925141	−0.154326033
Acetate-producing bacteria	0.039344707	0.220354319	−0.2428482	0.086927195	−0.105764379
Propionate-producing bacteria	−0.452729053	0.226588779	−0.03027371	0.363779549	0.251903984
Proteolytic bacteria	0.210227579	0.49353678	−0.2307229	0.128305744	0.393570457

Color scale interpretation: red shades indicate increasingly negative correlations (r < 0), whereas green shades represent increasingly positive correlations (r > 0). Yellow corresponds to values close to zero, indicating absence of correlation.

## Data Availability

The original contributions presented in this study are included in the article. Further inquiries can be directed to the corresponding author.

## Abbreviations

The following abbreviations are used in this manuscript:

DNA	Deoxyribonucleic Acid
FEV1	Forced Expiratory Volume in 1 Second
FVC	Forced Vital Capacity
GINA	Global Initiative for Asthma
IQR	Interquartile Range
LPS	Lipopolysaccharides
NGS	Next-Generation Sequencing
PCR	Polymerase Chain Reaction
REDCap	Research Electronic Data Capture
SCFA	Short-Chain Fatty Acids
rRNA	Ribosomal Ribonucleic Acid
KIDMED	Mediterranean Diet Quality Index for children and adolescents
